# Crystallization and Morphology of Triple Crystalline Polyethylene-*b*-poly(ethylene oxide)-*b*-poly(ε-caprolactone) PE-*b*-PEO-*b*-PCL Triblock Terpolymers

**DOI:** 10.3390/polym13183133

**Published:** 2021-09-16

**Authors:** Eider Matxinandiarena, Agurtzane Múgica, Manuela Zubitur, Viko Ladelta, George Zapsas, Dario Cavallo, Nikos Hadjichristidis, Alejandro J. Müller

**Affiliations:** 1 POLYMAT and Department of Polymers and Advanced Materials: Physics, Chemistry and Technology, University of the Basque Country UPV/EHU, Paseo Manuel Lardizábal 3, 20018 Donostia-San Sebastián, Spain; eider.matxinandiarena@ehu.eus (E.M.); agurtzane.mugica@ehu.eus (A.M.); 2 Department of Chemical and Environmental Engineering, University of the Basque Country UPV/EHU, Plaza Europa 1, 20018 Donostia-San Sebastián, Spain; manuela.zubitur@ehu.eus; 3 Polymer Synthesis Laboratory, KAUST Catalysis Center, Physical Sciences and Engineering Division, King Abdullah University of Science and Technology (KAUST), Thuwal 23955, Saudi Arabia; viko.ladelta@kaust.edu.sa (V.L.); georgios.zapsas@kaust.edu.sa (G.Z.); 4 Department of Chemistry and Industrial Chemistry, University of Genova, via Dodecaneso 31, 16146 Genova, Italy; dario.cavallo@unige.it; 5 Ikerbasque, Basque Foundation for Science, Plaza Euskadi 5, 48009 Bilbao, Spain

**Keywords:** triblock terpolymers, polyethylene (PE), poly(ethylene oxide) (PEO), poly(ɛ-caprolactone) (PCL), tricrystalline spherulites

## Abstract

The morphology and crystallization behavior of two triblock terpolymers of polymethylene, equivalent to polyethylene (PE), poly (ethylene oxide) (PEO), and poly (ε-caprolactone) (PCL) are studied: PE_22_^7.1^-*b*-PEO_46_^15.1^-*b*-PCL_32_^10.4^ (T1) and PE_37_^9.5^-*b*-PEO_34_^8.8^-*b*-PCL_29_^7.6^ (T2) (superscripts give number average molecular weights in kg/mol and subscripts composition in wt %). The three blocks are potentially crystallizable, and the triple crystalline nature of the samples is investigated. Polyhomologation (C1 polymerization), ring-opening polymerization, and catalyst-switch strategies were combined to synthesize the triblock terpolymers. In addition, the corresponding PE-*b*-PEO diblock copolymers and PE homopolymers were also analyzed. The crystallization sequence of the blocks was determined via three independent but complementary techniques: differential scanning calorimetry (DSC), in situ SAXS/WAXS (small angle X-ray scattering/wide angle X-ray scattering), and polarized light optical microscopy (PLOM). The two terpolymers (T1 and T2) are weakly phase segregated in the melt according to SAXS. DSC and WAXS results demonstrate that in both triblock terpolymers the crystallization process starts with the PE block, continues with the PCL block, and ends with the PEO block. Hence triple crystalline materials are obtained. The crystallization of the PCL and the PEO block is coincident (i.e., it overlaps); however, WAXS and PLOM experiments can identify both transitions. In addition, PLOM shows a spherulitic morphology for the PE homopolymer and the T1 precursor diblock copolymer, while the other systems appear as non-spherulitic or microspherulitic at the last stage of the crystallization process. The complicated crystallization of tricrystalline triblock terpolymers can only be fully grasped when DSC, WAXS, and PLOM experiments are combined. This knowledge is fundamental to tailor the properties of these complex but fascinating materials.

## 1. Introduction

Crystallization in block copolymers is a subject widely studied in the past decades [1,2,3,4,5,6,7,8,9,10,11]. It is vital to understand the morphology upon crystallization since it is directly related to the final properties of a material. Many applications can take advantage of these materials due to the different chemical nature of the segments that form a block copolymer [9,12,13,14]. In addition, many other factors such as composition, molecular weight, crystallization protocol, segregation strength, and block miscibility affect the crystallization behavior. As different morphologies can be developed, the final performance of the materials can be tuned by varying these factors [2,4,7,9,11,15,16,17,18].

AB-type diblock copolymers with one or two crystallizable blocks have been studied in the past few decades. Among medium or strongly segregated systems, the diblock copolymer PE-*b*-PLLA [18,19,20,21,22,23,24] is a well-known system. Müller et al. [19,20,21] reported strong segregation strength for these diblock copolymers and a lamellar morphology for compositions close to 50/50. Therefore, they did not see any spherulitic-type morphology as expected. When the content of PLLA in the diblock is between 89 and 96%, then spherulitic morphologies have been reported in the literature, as PLLA conforms the matrix phase [25]. The overall crystallization rate of both PLLA block and PE block in the diblock copolymers [19,20,21] was slower than that of the corresponding PLLA and PE homopolymers. In addition, coincident crystallization occurs, since the crystallization transitions of the PE block and the PLLA block overlap employing cooling rates higher than 2 °C/min.

Other double crystalline diblock copolymers show miscible or weakly segregated behaviors, and several studies have been reported about the crystallization process of these systems [7,26,27,28,29,30,31,32,33], although the most relevant ones are: PEO-*b*-PCL [7,34,35,36,37,38,39,40,41,42,43,44,45,46,47,48,49,50,51], PEO-*b*-PLLA [52,53,54,55,56,57,58,59,60,61,62,63,64,65], and PCL-*b*-PLA [29,30,32,66,67,68,69,70,71,72,73,74], because of their possible applications in the biomedical field due to the biodegradable and biocompatible nature of the blocks [14,75,76,77,78]. Additionally, some ABA-type systems have also been analyzed, such as PBT-*b*-PEO-*b*-PBT [79], PEO-*b*-PEB-*b*-PEO [80], or PLLA-*b*-PVDF-*b*-PLLA [81], for instance. The addition of a third potentially crystallizable block to diblock copolymers results in a more complex analysis of the crystallization behavior. Few studies have been published about tricrystalline triblock terpolymers, such as ABC-type triblock terpolymers and ABCBA pentablock terpolymers, including the apolar PE block, and the polar PEO, PCL, and PLLA blocks [17,24,37,42,82,83,84,85,86,87,88,89,90,91,92,93,94,95,96].

Palacios et al. studied the crystallization and morphology of ABC triblock terpolymers with three crystallizable blocks: PEO, PCL, and PLLA [92,93,94,95]. They [92] highlighted the triple crystalline nature of the PEO-*b*-PCL-*b*-PLLA triblock terpolymer, with the three different blocks crystallizing independently upon cooling from the melt. Even when changing the PLLA content, crystallization of the blocks follows this sequence: the PLLA block first, the PCL block second, and finally the PEO block. Melt miscibility of the three blocks was confirmed by SAXS. In addition, PLOM experiments showed that the first crystallized PLLA block determines the final morphology since the PCL block and the PEO block crystallized within the interlamellar regions of the PLLA templated spherulites, maintaining the superstructure determined by the PLLA block and forming triple crystalline spherulites. The crystallization of the PCL and the PEO blocks was evidenced by a change in the birefringence. There are several examples of confined crystallization of one block within the lamellae of another previously crystallized block [1,7,97,98].

Furthermore, by SAXS and AFM experiments, Palacios et al. [94] were able to identify a trilamellar self-assembly with lamellae of the three blocks at room temperature. Based on extensive observations and SAXS simulations, they proposed an alternation of single lamellae of PEO or PCL in between two PLLA lamellae. Very few reports have been published about the crystalline morphology in AB diblock copolymers and ABC triblock terpolymers from the melt by in situ AFM, and only two blocks crystallized in those samples [99,100]. Palacios et al. [17] analyzed by in situ hot-stage AFM the evolution of the trilamellar morphology upon melting of the PEO-*b*-PCL-*b*-PLLA triblock terpolymer. Three different lamellar populations were detected at different temperatures; the melting of each of the populations gives information about the corresponding block: the thinnest lamellae corresponded to the PEO block (the first block to melt at 45 °C), the medium size lamellae to the PCL block (melted at 60 °C), and the thickest lamellae to the highest melting temperature block, i.e., PLLA.

Still, few works have been published using the apolar PE block as one of the crystallizable blocks in triblock terpolymers. Müller et al. [96] analyzed the crystalline behavior and morphology of PE-*b*-PEO-*b*-PLLA and PE-*b*-PCL-*b*-PLLA triblock terpolymers employing different cooling rates. DSC, WAXS, and PLOM techniques were used to confirm the triple crystalline character of the copolymers. They concluded that there is no change in the sequential crystallization for the PE_21_^2.6^-*b*-PEO_32_^4.0^-*b*-PLLA_47_^5.9^ triblock terpolymer using 1 or 20 °C/min, since the sequence remains the following: the PE block crystallizes first, then the PLLA block, and finally the PEO block. However, the crystallization sequence changed in the PE_21_^7.1^-*b*-PCL_12_^4.2^-*b*-PLLA_67_^23.0^ triblock terpolymer, since when using 20 °C/min as cooling rate, the crystallization begins with the PE block. In contrast, at 1 °C/min the PLLA is the first block to crystallize. PLOM experiments showed that this variation in the crystallization sequence affects the final morphology, so the cooling rate is a factor that can be used to tune the final properties.

In the present work, the triple crystalline nature of PE-*b*-PEO-*b*-PCL triblock terpolymers is analyzed, varying molecular weight and block content. The corresponding PE-*b*-PEO diblock copolymers and PE homopolymers are also investigated. Samples were synthesized by combining polyhomologation and catalyst-switch strategies. We study the influence of molecular weight and block composition on the crystallization of these triblock terpolymers, consisting in an apolar (PE) and two polar blocks, PEO (biocompatible) and PCL (biodegradable). The study employs differential scanning calorimetry (DSC), in situ small-angle and wide-angle X-ray scattering (SAXS/WAXS), and polarized light optical microscopy (PLOM). Understanding the crystalline behavior and the analysis of the morphology is essential to tune crystallinity and obtain novel materials with enhanced properties.

## 2. Materials and Methods

### 2.1. Materials

**Scheme 1:**
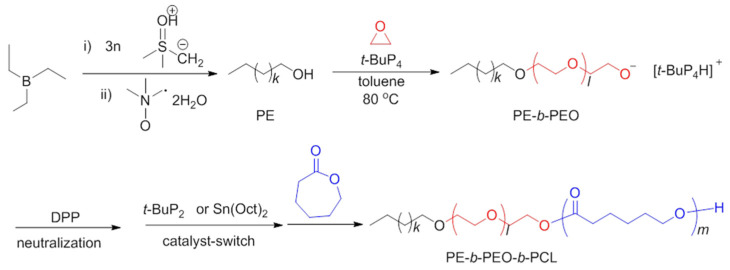
Synthesis of tricrystalline terpolymer PE-*b*-PEO-*b*-PCL by a combination of polyhomologation and two catalyst-switch strategies [102].

All reagents used for the synthesis of the triblock terpolymers were purchased from Merck KGaA (Darmstadt, Germany). Two different “catalyst switch” strategies were used in the synthesis of the tricrystalline terpolymers poly (ethylene)-*b*-poly (ethylene oxide)-*b*-poly (ε-caprolactone) (PE-*b*-PEO*-b*-PCL). First, the polyhomologation of dimethylsulfoxonium methylide was performed to synthesize a hydroxyl-terminated polyethylene (PE-OH) macroinitiator [101]. Then, the strong phospazene base *t*-BuP_4_ was employed as the catalyst to promote the ring-opening polymerization (ROP) of ethylene oxide (EO) to obtain PE-*b*-PEO, followed by the addition of diphenyl phosphate (DPP) to neutralize *t*-BuP_4_. For the ROP of *ε*-caprolactone (CL), two different catalysts were used, Sn(Oct)_2_ for T1 (organic/metal catalyst-switch), and phosphazene base *t*-BuP_2_ for T2 (organic/organic catalyst-switch). These catalyst switch-strategies were applied to avoid as many possible side-reactions during the ROP of CL in toluene at 80 °C (Scheme 1) [102].

Table 1 shows the molecular weights of each of the blocks of the synthesized triblock terpolymers. The subscript numbers represent composition in wt %, and superscripts indicate *M_n_* values of each block in kg/mol. The polyethylene block precursors are not 100% linear because of possible side reactions and monomer purity issues. NMR measurements indicate that the PE block of T1 (see Table 1) contains 0.32% propyl side groups and 3% methyl groups, and that of T2 contains 0.45% propyl side groups and 2% methyl groups. Different melting points are obtained because of this variation in microstructure, since the *T_m_* value of PE^7.1^ is 129.7 °C, while that of PE^9.5^ is 117 °C (see Table S3), as the latter contains a higher amount of short-chain branches.

The formation of double crystalline copolymers and triple crystalline terpolymers was confirmed by differential scanning calorimetry (DSC), polarized light optical microscopy (PLOM), and X-ray diffraction (SAXS/WAXS).

### 2.2. Methods

#### 2.2.1. Differential Scanning Calorimetry (DSC)

Non-isothermal DSC experiments were carried out with a Perkin Elmer DSC Pyris 1 (Perkin Elmer, Norwalk, USA) equipped with a refrigerated cooling system (Intracooler 2P). Indium and tin standards were used for the calibration of the equipment. Aluminum pans with about 3 mg of sample were tested using ultra-high quality nitrogen atmosphere.

A temperature range between 0 and 160 °C and 20 °C/min as cooling and heating rates were employed in non-isothermal DSC experiments. The samples are kept for 3 min 30 °C above the peak melting temperature of the block showing the highest melting temperature to erase the thermal history of the samples. They are then cooled down at 20 °C/min keeping them 1 min at low temperatures, and finally heating up also at 20 °C/min until the block at the highest temperature melts.

#### 2.2.2. Small-Angle and Wide-Angle X-ray Scattering (SAXS/WAXS)

Simultaneous in situ small-angle X-ray scattering (SAXS) and wide-angle X-ray scattering (WAXS) experiments were performed at the ALBA Synchrotron facility in Barcelona (Spain), beamline BL11-NCD. A Linkam THMS600 (Linkam, Surrey, UK) hot stage coupled to a liquid nitrogen cooling system was used to cool and heat the samples, which were previously placed into glassy capillaries. The same thermal protocol adopted in the non-isothermal DSC experiments was used to get the SAXS/WAXS patterns, in which crystallization and melting of the samples are followed, thus obtaining comparable results by the two different techniques.

The X-ray energy source was 12.4 keV (*λ* = 1.03 Å). For the SAXS setup, the distance between the sample and the detector (ADSC Q315r detector, Poway, CA, USA, with a resolution of 3070 × 3070 pixels, pixel size of 102 µm^2^) was 6463 mm with a tilt angle of 0°. Calibration was performed with silver behenate. Regarding WAXS configuration, a distance of 132.6 mm was used between the sample and the detector, with a tilt angle of 21.2°. Chromium (III) oxide (Rayonix LX255-HS detector, Evanston, IL, USA, with a resolution of 1920 × 5760 pixels, pixel size of 44 µm^2^) was employed for calibration. Scattering intensity as a function of scattering vector, q = 4πsinθλ^−1^ data are obtained, where *λ* is the X-ray wavelength, and 2*θ* is the scattering angle.

#### 2.2.3. Polarized Light Optical Microscopy (PLOM)

An Olympus BX51 polarized light optical microscope (Olympus, Tokyo, Japan) was used to follow the morphological changes occurring within the samples while cooled and heated at a constant rate of 20 °C/min. For accurate temperature control, a Linkam THMS600 (Linkam, Surrey, UK) hot stage with liquid nitrogen was used. Micrographs were recorded by an Olympus SC50 camera (Olympus, Tokyo, Japan). First, a glass slide in which samples are melted is used, with a glass coverslip, and then, 20 °C/min as cooling and heating rates are employed. Morphological variations that occur during the application of this constant rate are recorded as micrographs in which crystallization and melting of each of the blocks can be followed.

Furthermore, the software ImageJ [103] was used to analyze the micrographs by measuring transmitted light intensities. The increase in light intensity detected refers to the increase in crystal content of a certain sample since crystallization of one component has started. Crystallization of this component can be followed by the increase in intensity by decreasing temperature, and the temperature range at which crystallization of a specific block occurs can be determined. In order to detect intensity changes, the whole micrographs are considered as “region of interest”. Thus, all superstructures that can be formed during the cooling scans contribute to this analysis. So, the entire crystallization process is followed by analyzing intensity changes as a function of temperature, and the crystallization temperature of a particular block of the diblock copolymers and triblock terpolymers can be determined.

## 3. Results and Discussion

### 3.1. Small-Angle X-ray Scattering (SAXS)

**Figure 1:**
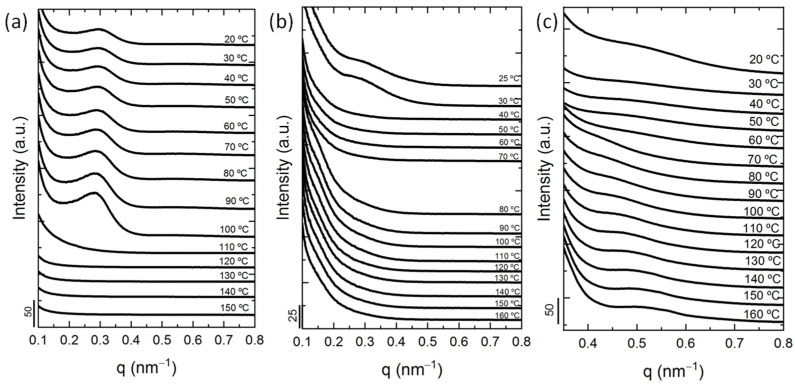
SAXS ramp down patterns at 20 °C/min for (**a**) PE^7.1^, (**b**) PE_32_^7.1^-*b*-PEO_68_^15.1^, and (**c**) PE_22_^7.1^-*b*-PEO_46_^15.1^-*b*-PCL_32_^10.4^ (T1) at the indicated temperatures.

SAXS measurements are useful to study not only the phase segregation in the melt but also if the phase segregation is kept when the block components crystallize or if crystallization destroys it by breaking out the phase structure of the melt. Figure 1 shows the SAXS patterns of the homopolymer PE^7.1^, the diblock copolymer PE_32_^7.1^-*b*-PEO_68_^15.1^, and the triblock terpolymer PE_22_^7.1^-*b*-PEO_46_^15.1^-*b*-PCL_32_^10.4^ (T1) upon cooling from the melt.

For the homopolymer PE^7.1^ and the diblock copolymer PE_32_^7.1^-*b*-PEO_68_^15.1^ (Figure 1a,b), there is no phase segregation in the melt, as evidenced by the lack of scattering peaks in the molten state. The broad peak that appears at lower temperatures corresponds to the diffraction from crystalline lamellar stacks in the formed superstructures (i.e., spherulites or axialites).

However, there is weak phase segregation for the PE_22_^7.1^-*b*-PEO_46_^15.1^-*b*-PCL_32_^10.4^ (T1) triblock terpolymer (Figure 1c) since there is a broad scattering peak in the melt, which disappears as crystallization breaks out when the first block upon cooling from the melt starts to crystallize (i.e., the PE block). This behavior is evidenced by the shift in *q* values between the reflection in the melt and the weaker reflection at room temperature, which appears at lower *q* values. The broad peak at room temperature corresponds to the average long period of the lamellae formed during the crystallization process because the phase structure established by phase segregation in the melt was destroyed by the break-out.

**Figure 2:**
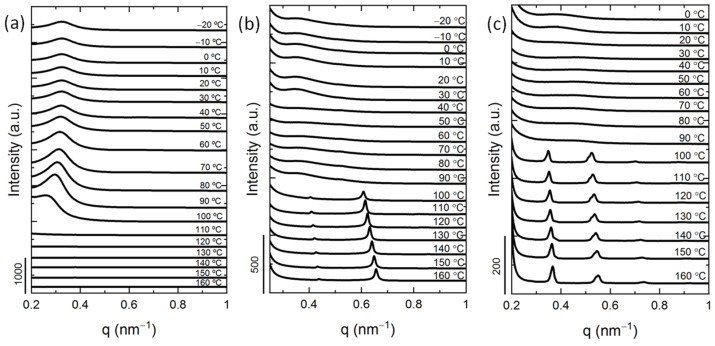
SAXS ramp down patterns at 20 °C/min for (**a**) PE^9.5^, (**b**) PE_52_^9.5^-*b*-PEO_48_^8.8^, and (**c**) PE_37_^9.5^-*b*-PEO_34_^8.8^-*b*-PCL_29_^7.6^ (T2) at the indicated temperatures.

Figure 2 shows SAXS patterns of the homopolymer PE^9.5^, the diblock copolymer PE_52_^9.5^-*b*-PEO_48_^8.81^, and the triblock terpolymer PE_37_^9.5^-*b*-PEO_34_^8.8^-*b*-PCL_29_^7.6^ (T2) at the indicated temperatures reached upon cooling. In this case, the behavior of the homopolymer PE^9.5^ (Figure 2a) is the same as for the homopolymer PE^7.1^ (Figure 1a) explained above, not showing any phase segregation in the melt, as expected for a homopolymer.

The diblock copolymer PE_52_^9.5^-*b*-PEO_48_^8.81^ (Figure 2b) and the triblock terpolymer PE_37_^9.5^-*b*-PEO_34_^8.8^-*b*-PCL_29_^7.6^ (T2) (Figure 2c) are phase segregated in the melt, with possible lamellar and interpenetrated morphologies, respectively, although more detailed analysis of the scattering curves would be needed to ascertain the exact melt morphology. The clear scattering peaks in the molten state in these two materials corroborate the phase segregation behavior; however, their phase segregation is weak, since when the first block crystallizes upon cooling, i.e., the PE block at 100 °C, the phase structure is destroyed, the one generated by phase segregation in the melt, as deduced by the change in *q* values and intensities of the scattering peaks.

One way to predict the segregation strength in linear diblock copolymers is by multiplying the Flory-Huggins interaction parameter (*χ*) (evaluated at the interest temperature in the melt) by *N* (the total degree of polymerization). The estimation becomes more difficult in the case of triblock terpolymers. Different behaviors can be predicted depending on the segregation strength values. Values equal or lower to 10 indicates miscibility in the melt, between 10 and 30 weak phase segregation, between 30 and 50 intermediate segregation, and if values are higher than 50, the systems are strongly segregated. A rough approximation for each pair of blocks is reported in Table S1 (see Supporting Information), using the solubility parameters of PE, PEO, and PCL from the literature [60,104]. In this case, the predicted values suggest that at least the diblock copolymers should be strongly segregated, but the experimental SAXS findings indicate miscibility for PE_32_^7.1^-*b*-PEO_68_^15.1^ and weak segregation for the PE_52_^9.5^-*b*-PEO_48_^8.8^.

As the dominant behavior during crystallization is that of break out, the final morphology is that of crystalline lamellae arranged in superstructures like axialites or spherulites. Therefore, we will not explore in detail the morphology of the materials in the melt, as it is destroyed upon crystallization.

### 3.2. Non-Isothermal Crystallization by DSC

DSC cooling and heating scans of the homopolymers, diblock copolymers, and triblock terpolymers of the two systems (Table 1) are discussed in this section. In addition, all data obtained are collected in Tables S2–S4 (Supporting Information).

**Figure 3:**
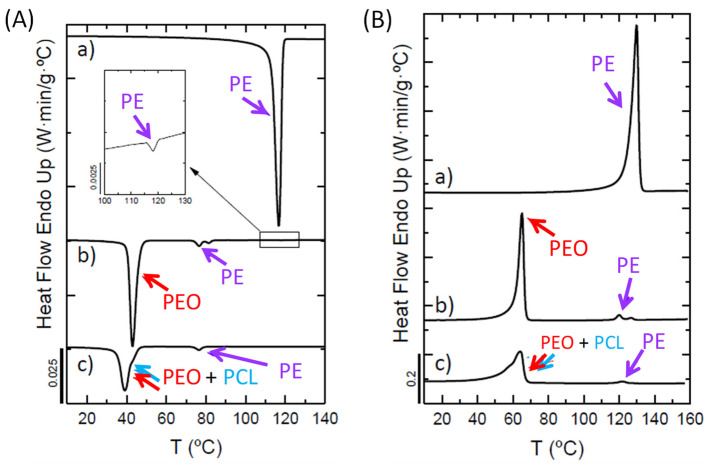
DSC scans at 20 °C/min for (a) PE^7.1^, (b) PE_32_^7.1^-*b*-PEO_68_^15.1^, and (c) PE_22_^7.1^-*b*-PEO_46_^15.1^-*b*-PCL_32_^10.4^ (T1) of (**A**) cooling from the melt, with a close-up to notice the very first crystallization exotherm of the PE block and (**B**) subsequent heating with arrows indicating transitions for each block.

Figure 3 shows the cooling (A) and heating (B) DSC scans for the PE^7.1^ homopolymer, PE_32_^7.1^-*b*-PEO_68_^15.1^ diblock copolymer, and PE_22_^7.1^-*b*-PEO_46_^15.1^-*b*-PCL_32_^10.4^ (T1) triblock terpolymer. The crystallization peak of each block (*T_c_*) has been assigned using WAXS data collected under identical conditions at the synchrotron (shown and described below). The same color code is used throughout this work to highlight the crystallization and melting of the different blocks (blue for PCL, red for PEO, and violet for PE). The sharp exotherm (Figure 3A(a)) and subsequent endotherm (Figure 3B(a)) of the neat PE^7.1^ precursor is a consequence of its linear character (synthesized by polyhomologation).

In the PE_32_^7.1^-*b*-PEO_68_^15.1^ diblock copolymer, PE (violet arrow) is the first block crystallizing upon cooling from the melt, and then the crystallization of the PEO block (red arrow) occurs (Figure 3Ab). The crystallization of the PE block does not occur in a unique step since three exothermic crystallization peaks appear for the PE block crystallization: at 118 °C, 82 °C, and 79 °C. This evidences that the PE block crystallizes in a fractionated way, which means that several crystallization exotherms appear at lower temperatures instead of a single crystallization exotherm corresponding to the PE block’s bulk crystallization temperature. Note that as shown in Figure 1b, this diblock copolymer shows miscibility in the melt, and as crystallization occurs from a homogeneous melt, as well as only having 32 wt % of PE block content and a relatively low molecular weight, the crystallization of the PE block is somehow hindered, as evidenced by its crystallization enthalpy value of 22 J/g (Table S2). However, the sharp crystallization exotherm of the PEO block and the high block content (68 wt %) suggest its high crystallization ability, as the enthalpy for the PEO is 177 J/g (Table S2).

The crystallization in the PE_22_^7.1^-*b*-PEO_46_^15.1^-*b*-PCL_32_^10.4^ (T1) triblock terpolymer (Figure 3A(c)) starts with the PE block (violet arrow). In this case, the PE block content is low (22 wt %), and a very small crystallization exotherm is observed in the cooling scan (14 J/g) (Table S2). Crystallization continues with the PCL block (blue arrow) and the PEO block (red arrow). Although the crystallization peaks of the PEO and the PCL blocks are overlapped, WAXS results below demonstrate that the PCL block crystallizes some degrees above the PEO block (Figure 5c). As we are not able to distinguish between both transitions, an estimation of the crystallization enthalpies is reported in Table S2 by employing block content for the calculations.

Figure 3B shows the subsequent heating scans with the endothermic melting peaks (*T_m_*) for each sample; data are collected in Table S3. The homopolymer PE^7.1^ (Figure 3B(a)) shows a crystallinity value of 75% (Table S4), as expected, observing the sharp melting transition. For the diblock copolymer (Figure 3B(b)), melting starts with the PEO block (red) with a crystallinity value of 85%; and it continues with the PE block melting (violet), with a crystallinity value of only 7% (Table S4), because as previously mentioned, small block content and cooling from a homogenous melt are not the best scenarios to enhance crystallization. The overlapped melting peak at the lowest temperature for the triblock terpolymer (Figure 3B(c)) corresponds to the PEO (red) and the PCL (blue) blocks (an estimation of the crystallinity values is provided in Table S4), whereas the melting at the highest temperatures occurs for the PE block crystals, although its crystallinity degree is only 5% (Table S4) of its 32 wt % block content in the terpolymer.

**Figure 4:**
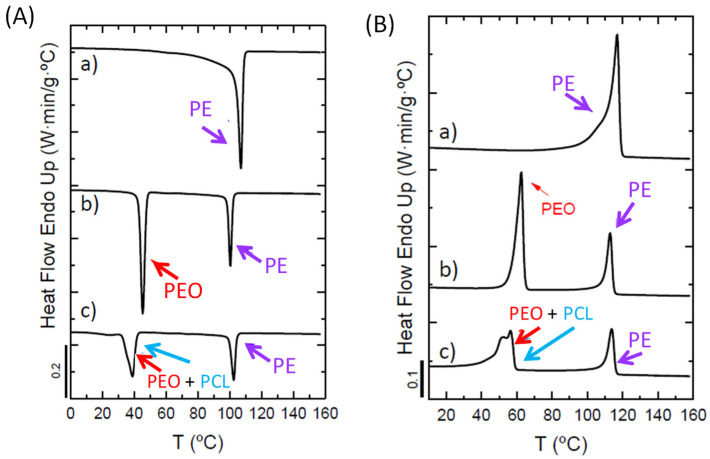
DSC scans at 20 °C/min for (a) PE^9.5^, (b) PE_52_^9.5^-*b*-PEO_48_^8.8^, and (c) PE_37_^9.5^-*b*-PEO_34_^8.8^-*b*-PCL_29_^7.6^ (T2) of (**A**) cooling from the melt and (**B**) subsequent heating with arrows indicating transitions for each block.

Figure 4 shows the cooling and heating scans of the PE^9.5^ homopolymer, the PE_52_^9.5^-*b*-PEO_48_^8.8^ diblock copolymer, and the PE_37_^9.5^-*b*-PEO_34_^8.8^-*b*-PCL_29_^7.6^ (T2) triblock terpolymer. The crystallization and melting transitions of the blocks in these samples (Figure 4A(c)–B(c)) follow the same trend described before in Figure 3, but with some differences due to the phase behavior of the materials.

The crystallization of PE^9.5^ homopolymer (Figure 4A(a)) occurs in a single and sharp transition. For the PE_52_^9.5^-*b*-PEO_48_^8.8^ diblock copolymer (Figure 4A(b)), the crystallization of the PE block (violet) occurs at high temperatures, followed by the crystallization of the PEO block (red) at lower temperatures. Note that the PE block crystallizes in a unique crystallization step in this diblock copolymer, not in a fractionated way as in the previous diblock copolymer discussed before (Figure 3A(b)). The difference remains in the phase behavior in the melt, on the one hand, since this diblock copolymer shows weak phase segregation (as evidenced by SAXS experiments shown in Figure 2b), and the fact of being segregated in the melt enhances the crystallization ability of the PE block. In addition, the PE block content is higher in this copolymer (52 wt %) with a higher molecular weight (9500 vs. 7100 g/mol). So, higher PE content and cooling from a segregated melt, do not largely hinder its crystallization, showing a crystallization enthalpy of 81 J/g (Table S2).

The crystallization sequence in the PE_37_^9.5^-*b*-PEO_34_^8.8^-*b*-PCL_29_^7.6^ (T2) triblock terpolymer is the same as the one explained in the previous triblock terpolymer (T1) (Figure 3A(c)): first the PE block (violet), and then the PCL (blue) and PEO (red) blocks. Although, also in this case, there is an overlap of the crystallization peaks of the PCL and PEO blocks, WAXS measurements show () that the PEO block crystallizes a few degrees lower than the PCL block; and estimations of the enthalpies are provided in Table S2.

The subsequent heating scans are shown in Figure 4B. The homopolymer PE^9.5^ in Figure 4B(a) shows a clear melting transition and a crystallinity value of 55% (Table S4). In the case of the PE_52_^9.5^ -*b*- PEO_48_^8.8^ diblock copolymer (Figure 4B(b)), the melting starts with the PEO block (red) and ends with the PE block (violet). As previously mentioned, segregation in the melt and higher PE content enhance its crystallization, and thus, a clear and sharp melting transition with a crystallinity value of 27% is obtained (Table S4). Finally, the PE_37_^9.5^-*b*-PEO_34_^8.8^-*b*-PCL_29_^7.6^ (T2) triblock terpolymer follows the same trend as in the triblock terpolymer T1 (Figure 3B(c)): melting of the PEO (red) and PCL (blue) blocks occur with a difference of some degrees, although not enough to distinguish between both DSC melting transitions (demonstrated by WAXS experiments in Figures S3(c)–S4(c)); and melting of the PE block showing a higher crystallinity degree (44%) (Table S3).

### 3.3. In Situ Wide Angle X-ray Scattering (WAXS) Real-Time Synchrotron Results

The crystallization of each block in the WAXS patterns is identified by analyzing the crystal planes indexing for the PE, PCL, and PEO blocks reported in Table S5 [29,32,38,60,64,66,92,105,106]. In addition, normalized intensity measurements as a function of temperature upon cooling from the melt (at 20 °C/min) are provided, confirming the samples’ double and triple crystalline nature.

**Figure 5:**
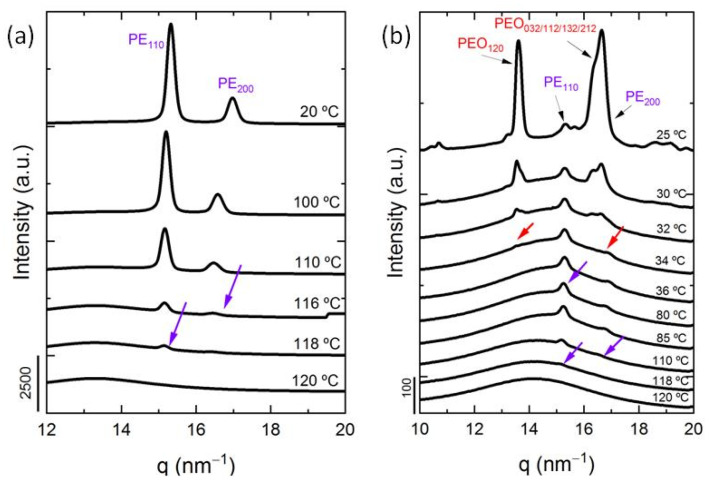

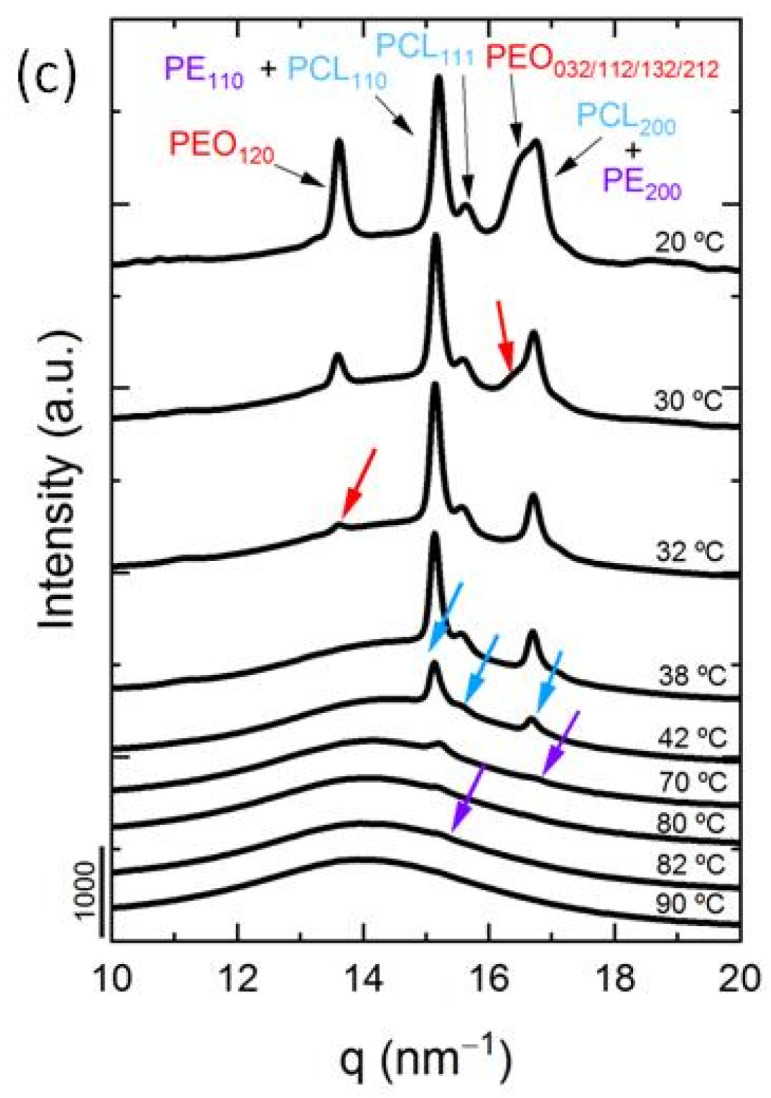
WAXS patterns upon cooling from the melt at 20 °C/min for (**a**) PE^7.1^, (**b**) PE_32_^7.1^-*b*-PEO_68_^15.1^, and (**c**) PE_22_^7.1^-*b*-PEO_46_^15.1^-*b*-PCL_32_^10.4^ (T1) at different temperatures with colored arrows indicating crystallization of each block and the (hkl) planes.

As shown in Figure 5, all blocks are able to crystallize, as demonstrated by the presence of their characteristic scattering peaks at certain *q* values, pointed out with the colors we are employing throughout the whole work.

**Figure 6:**
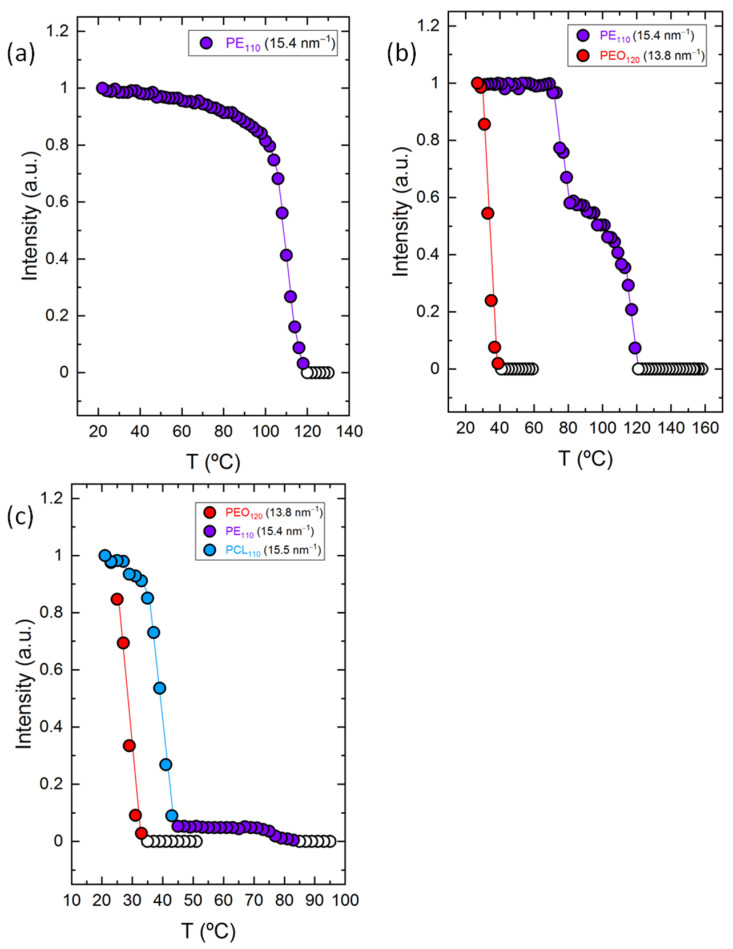
Normalized WAXS intensities as a function of temperature calculated from WAXS data represented in Figure 5 for (**a**) PE^7.1^ (PE_110_ (15.4 nm^−1^)), (**b**) PE_32_^7.1^-*b*-PEO_68_^15.1^ (PE_110_ (15.4 nm^−1^) and PEO_120_ (13.8 nm^−1^)), and (**c**) PE_22_^7.1^-*b*-PEO_46_^15.1^-*b*-PCL_32_^10.4^ (PEO_120_ (13.8 nm^−1^), PE_110_ (15.4 nm^−1^) and PCL_110_ (15.5 nm^−1^)) with colored data points and lines indicating crystallization of the corresponding blocks. Empty data points correspond to the molten state.

The PE^7.1^ homopolymer crystallization starts at 118 °C (Figure 5a), as its characteristic scattering peak at 15.4 nm^−1^ (violet arrow) corresponding to the (110) crystallographic plane appears at this temperature. Cooling down the sample, at 16.9 nm^−1^, the other scattering peak of the (200) plane confirms PE crystallization. In addition, the normalized WAXS intensity calculation as a function of temperature for the PE_110_ (15.4 nm^−1^) reflection in Figure 6a confirms the crystallization of the PE block by the sharp increase of the intensity.

Figure 5b shows that the first block to crystallize, during cooling from the melt, in the PE_32_^7.1^-*b*-PEO_68_^15.1^ diblock copolymer is PE at 118 °C (violet arrows) with its scattering peaks at 15.4 and 16.9 nm^−1^ (reflections (110) and (200), respectively). At lower temperatures, 34 °C, the PEO block (red arrows) starts to crystallize with its (120) and (032)/(112)/(132)/(212) reflections at 13.8 and 16.4 nm^−1^, respectively. Although the crystallization of these two blocks is clear, the normalized WAXS intensities calculated in Figure 6b, show this sequential crystallization by analyzing separately the unique scattering peaks of the PEO_120_ (13.8 nm^−1^) and the PE_110_ (15.4 nm^−1^). At high temperatures, the intensity starts to increase at 118 °C due to PE crystallization, and the second increase at 82 °C also corresponds to PE, because as reported in Figure 3A(b), PE crystallizes in two steps.

Figure 5c corresponds to the PE_22_^7.1^-*b*-PEO_46_^15.1^-*b*-PCL_32_^10.4^ (T1) triblock terpolymer. In this case, the crystallization sequence starts with the PE crystallization (violet arrows), as evidenced by the PE_110_ reflection at 82 °C and the PE_200_ reflection at 70 °C. One may find this crystallization temperature low for the PE block, but as discussed previously in Figure 3A(c), the PE content is low (22 wt %) and the crystallization enthalpy is 14 J/g. The next block that crystallizes is the PCL block (blue arrows). At 42 °C, the PCL_110_ (15.5 nm^−1^), PCL_111_ (15.6 nm^−1^), and PCL_200_ (16.7 nm^−1^) reflections prove the presence of PCL block crystals. The last block to crystallize upon cooling from the melt is the PEO block (red arrows). The presence of its scattering peak at 13.8 nm^−1^ corresponding to the (120) crystallographic plane at 32 °C confirms the crystallization. At lower temperatures, the other characteristic peak of PEO (16.4 nm^−1^) appears at 30 °C corresponding to the (032/112/132/212) plane (Figure 5c). 

The normalized intensities are analyzed to detect the exact temperature at which each of the blocks crystallizes (Figure 6c). The joint reflections of PE_110_ (15.4 nm^−1^) and PCL_110_ (15.5 nm^−1^) are used to determine their crystallization temperature ranges. The first slight change in intensity at 82 °C confirms PE crystallization (violet), barely noticeable due to the low content of the PE block in the terpolymer (22 wt %). Then, the sharp increase at 42 °C indicates the crystallization of the PCL block (blue). The single PEO_120_ (13.8 nm^−1^) reflection (along with the other PE and PCL reflections) confirms its crystallization by a sharp increase in intensity.

**Figure 7:**
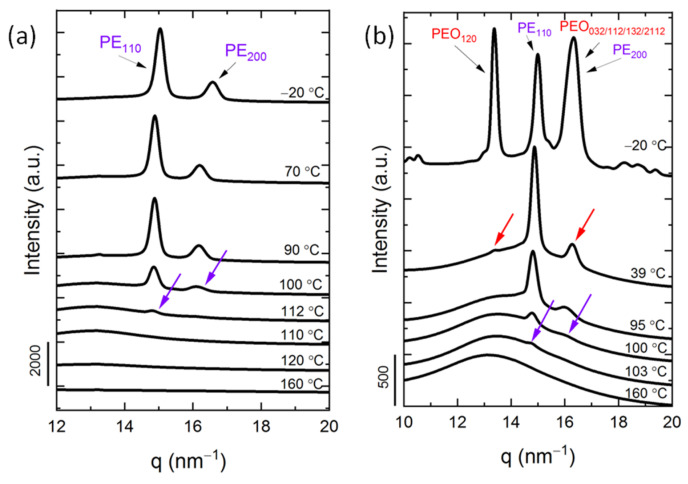

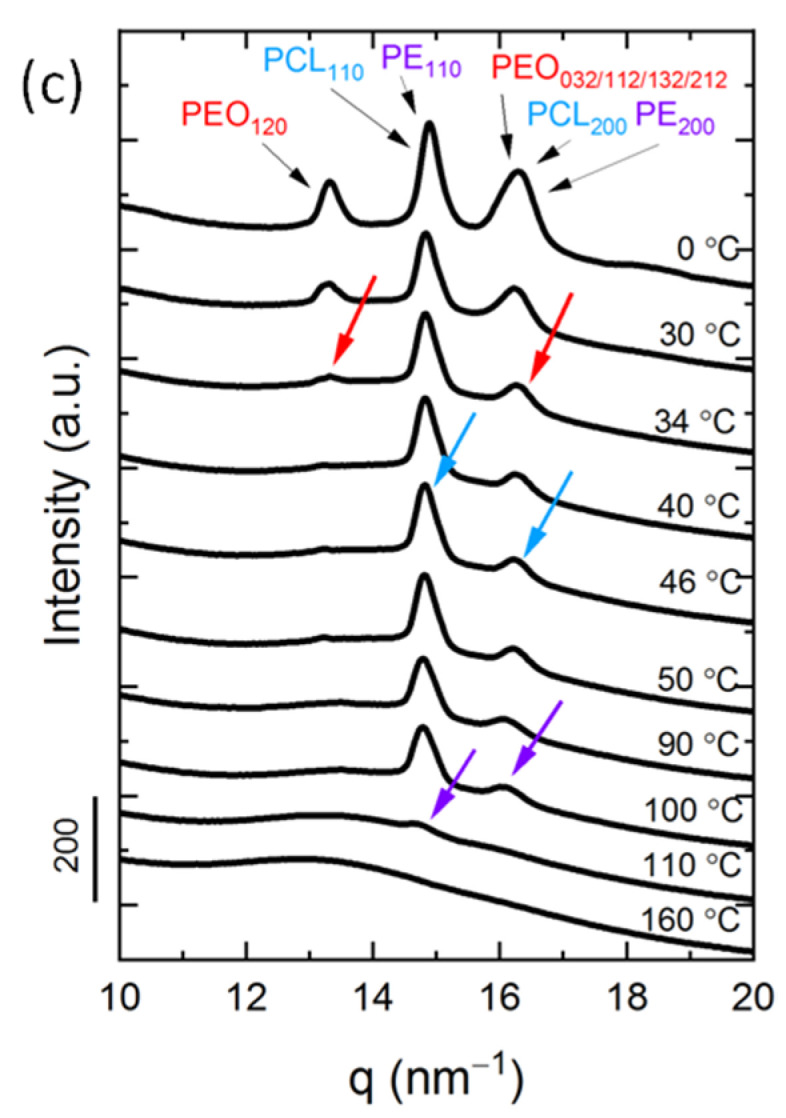
WAXS patterns upon cooling from the melt at 20 °C/min for (**a**) PE^9.5^, (**b**) PE_52_^9.5^-*b*-PEO_48_^8.8^, and (**c**) PE_37_^9.5^-*b*-PEO_34_^8.8^-*b*-PCL_29_^7.6^ (T2) at different temperatures with colored arrows indicating crystallization of each block and (hkl) planes.

**Figure 8:**
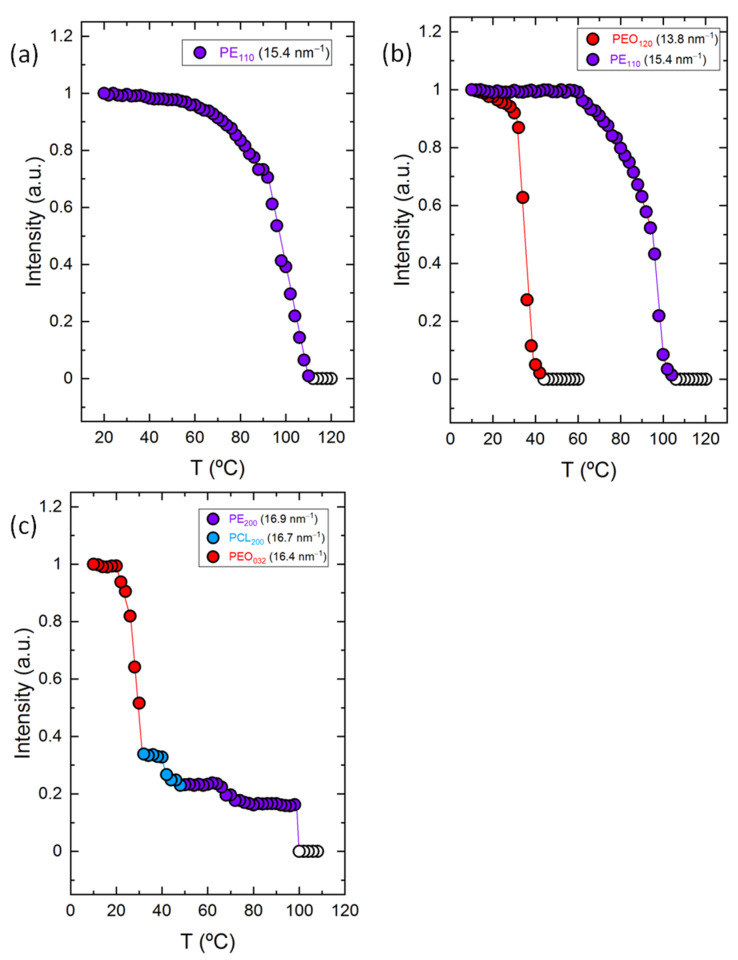
Normalized WAXS intensities as a function of temperature calculated from WAXS data represented in Figure 7 for (**a**) PE^9.5^ (PE_110_ (15.4 nm^−1^)), (**b**) PE_52_^9.5^-*b*-PEO_48_^8.8^ (PE_110_ (15.4 nm^−1^) and PEO_120_ (13.8 nm^−1^)), and (**c**) PE_37_^9.5^-*b*-PEO_34_^8.8^-*b*-PCL_29_^7.6^ (PE_200_ (16.9 nm^−1^), (PCL_1200_ (16.7nm^−1^) and (PEO_032_ (16.4 nm^−1^)) with colored data points and lines indicating crystallization of the corresponding blocks. Empty data points correspond to the molten state.

Similarly, in Figure 7 and Figure 8, WAXS patterns upon cooling the melt (at 20 °C/min) and the normalized intensity measurements confirm crystallization of all blocks in the other set of samples listed in Table 1: the homopolymer PE^9.5^, the diblock copolymer PE_52_^9.5^-*b*-PEO_48_^8.8^, and the triblock terpolymer PE_37_^9.5^-*b*-PEO_34_^8.8^-*b*-PCL_29_^7.6^ (T2).

In this case, Figure 7a shows that the crystallization of the homopolymer PE^9.5^ starts at 112 °C (PE_110_ at 15.4 nm^−1^), and the second scattering peak appears at 100 °C, PE_200_ (16.9 nm^−1^) (see violet arrows). Figure 8a shows the broad temperature range at which PE crystallizes since a plateau is not reached until approximately 60 °C, determining this way that PE crystallizes in between 112 and 60 °C.

Continuing with Figure 7b, the first reflection at 103 °C ((110) reflection at 15.4 nm^−1^) corresponds to the PE block, along with the (200) reflection (16.9 nm^−1^) at 100 °C (see violet arrows). The second block to crystallize in this diblock copolymer at 39 °C is the PEO block (red arrows), identified due to the presence of the (120) reflection at 13.8 nm^−1^ and ((032)/(112)/(132)/(212) reflections at 16.4 nm^−1^. Once again, normalized intensities in Figure 8b confirm the temperature ranges at which both the PE and the PEO blocks start to crystallize due to the sharp increase in the intensity of the corresponding peaks.

To conclude, Figure 7c shows the WAXS patterns for the PE_37_^9.5^-*b*-PEO_34_^8.8^*-b* PCL_29_^7.6^ (T2) triblock terpolymer. The crystallization sequence remains the same as in the previous triblock terpolymer discussed above (Figure 5c): the PE block first (violet arrows) at 110 °C ((110) and (200) reflections at 15.4 and 16.9 nm^−1^); then the PCL block (blue arrows) at 46 °C ((110) and (200) reflections at 15.5 and 16.7 nm^−1^); and finally, the PEO block (red arrows) at 34 °C ((120) and (032)/(112)/(132)/(212) reflections at 13.8 and 16.4 nm^−1^). In addition, the normalized intensities shown in Figure 8c demonstrate the crystallization of the three blocks by analyzing the joint reflection that the three blocks show at *q* values between 16.4 and 16.9 nm^−1^. Note that as the PE content is higher in this triblock terpolymer (T2) (37 wt% vs. 22 wt%), the increase in intensity is clearer than in the previous triblock terpolymer (T1), in which it was very low (Figure 6c).

In addition, to confirm the crystallization of every single block in the cooling scans, results for the subsequent heating scans are shown in the Supporting Information. Figures S1–S4 report WAXS diffraction patterns and normalized intensity measurements of both triblock terpolymers here analyzed (T1 and T2).

### 3.4. Polarized Light Optical Microscopy (PLOM) Observations

**Figure 9:**
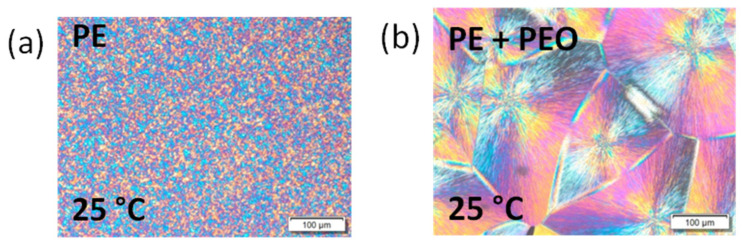
PLOM micrographs taken at room temperature after cooling from the melt at 20 °C/min for (**a**) PE^7.1^ and (**b**) PE_32_^7.1^-*b*-PEO_68_^15.1^, indicating the crystalline phases at 25 °C.

**Figure 10:**
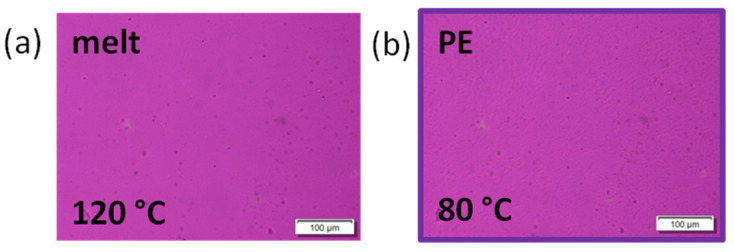

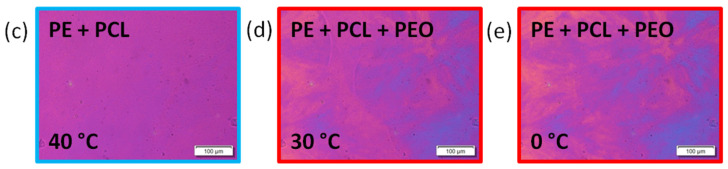
PLOM micrographs of the triblock terpolymer PE_22_^7.1^-*b*-PEO_46_^15.1^-*b*-PCL_32_^10.4^ (T1) cooling the sample from the melt at 20 °C/min. Colored squares (violet for PE, blue for PCL, and red for PEO) refer to the crystallized block at their corresponding temperature, indicated at the top of the micrographs for (**a**) molten state at 120 °C, (**b**) PE at 80 °C, (**c**) PE and PCL at 40 °C, (**d**) PE, PCL and PEO at 30 °C, and (**e**) PE, PCL, and PEO at 0 °C.

**Figure 11:**
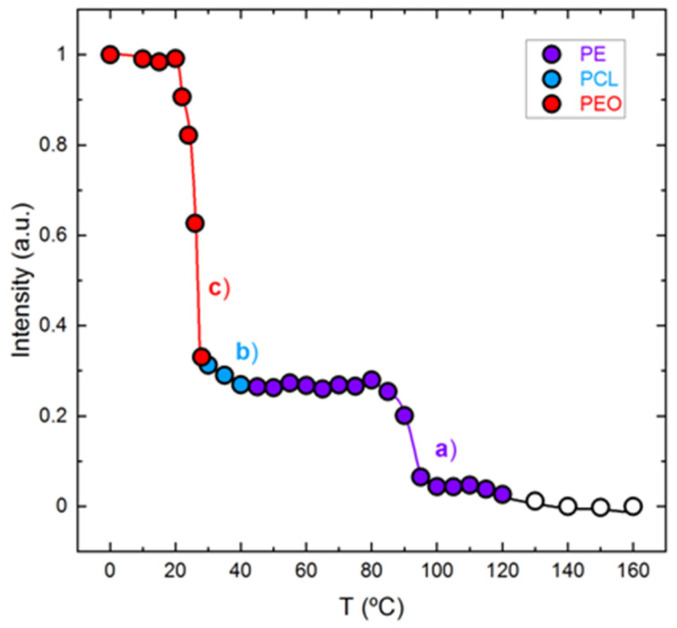
PLOM intensity measurement calculation from data in Figure 10 as a function of temperature during cooling from the melt at 20 °C/min, showing crystallization of (a) the PE block, (b) the PCL block, and (c) the PEO block for the triblock terpolymer PE_22_^7.1^-*b*-PEO_46_^15.1^-*b*-PCL_32_^10.4^ (T1). Colored data points and lines (violet for PE, blue for PCL, and red for PEO) are employed in order to follow the crystallization of the blocks. Empty data points correspond to the molten state of the sample.

**Figure 12:**
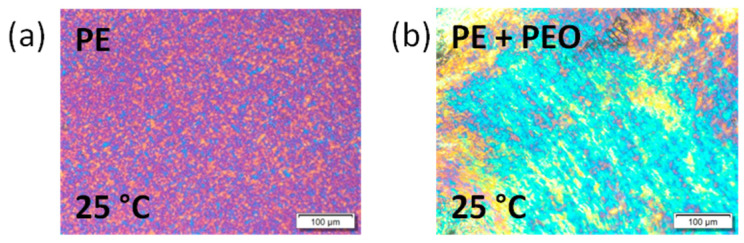
PLOM micrographs taken at room temperature after cooling the samples from the melt at 20 °C/min for (**a**) PE^9.5^ and (**b**) PE_52_^9.5^-*b*-PEO_48_^8.8^, indicating the crystallized blocks at room temperature.

PLOM was employed to follow crystallization of the blocks and to give evidence of the final morphology. Micrographs taken at room temperature (after cooling the samples at 20 °C/min) are shown in Figure 9, Figure 10, Figure 11 and Figure 12.

Figure 9a corresponds to the homopolymer PE^7.1^, showing very small spherulites. In Figure 9b, the PE_32_^7.1^-*b*-PEO_68_^15.1^ diblock copolymer shows large spherulites characteristic of PEO. According to the evidence gathered in the previous sections, the PE block crystallizes first, probably forming microspherulites that are later engulfed by the much larger PEO block spherulites.

The triple crystalline morphology of the PE_22_^7.1^-*b*-PEO_46_^15.1^-*b*-PCL_32_^10.4^ (T1) triblock terpolymer is shown in Figure 10, in which the whole cooling process at 20 °C/min was followed. Figure 10a indicates that the sample at 120 °C is in the molten state. Cooling to 80 °C (Figure 10b), the first block to start to crystallize is the PE block, forming very small and barely observable microspherulites. Due to this difficulty, light intensity measurements as a function of temperature were measured since slight changes in the PLOM micrographs can be better detected.

Figure 11 shows all intensity changes that occur during the cooling scan of this sample. Curve a of Figure 11 shows the increase in intensity related to the crystallization of the PE block, which crystallizes until saturation at 80 °C. Going back to Figure 10c, the second block to crystallize is the PCL block at 40 °C. A slight change is appreciable in this micrograph, but the difference in intensity in curve b of Figure 11 confirms the PCL block crystallization. Finally, Figure 10d,e shows the crystallization of the PEO block, which corresponds to the sharp increase in intensity in curve c of Figure 11. Due to the crystallization of the three blocks, a triple crystalline block copolymer is obtained.

The micrographs taken during the subsequent heating of this PE_22_^7.1^-*b*-PEO_46_^15.1^*-b* PCL_32_^10.4^ (T1) triblock terpolymer are provided in Figure S5 in the SI, along with the normalized intensity calculations as a function of temperature also in the SI (Figure S6). These graphs show the melting of all blocks, demonstrating the triple crystalline behavior of the sample. In addition, all PLOM observations match very well with DSC (Figure 3) and WAXS (Figure 5 and Figure 6) results previously discussed.

Regarding the second system listed in Table 1, the same PLOM observations were performed in order to compare the crystalline behavior of both series of samples. Figure 12 shows the PLOM micrographs at 25 °C of the precursors of the PE_37_^9.5^-*b*-PEO_34_^8.8^-*b*-PCL_29_^7.6^ (T2) triblock terpolymer after cooling the samples at a constant rate of 20 °C/min. Figure 12a corresponds to the PE^9.5^ homopolymer, in which very small PE spherulites can be observed. The micrograph in Figure 12b, on the contrary, refers to the PE_52_^9.5^-*b*-PEO_48_^8.8^ diblock copolymer. Although there are no clear PEO spherulites, it shows a double crystalline morphology at room temperature.

**Figure 13:**
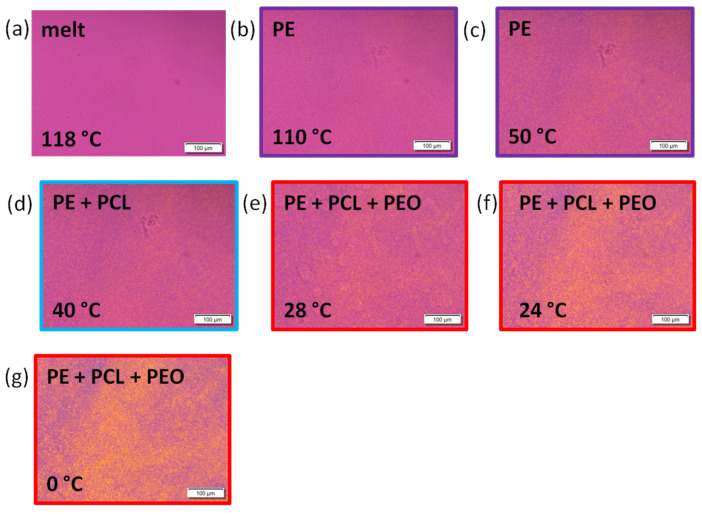
PLOM micrographs of the triblock terpolymer PE_37_^9.5^-*b*-PEO_34_^8.8^-*b*-PCL_29_^7.6^ (T2) cooling the sample from the melt at a constant rate of 20 °C/min. Colored squares (violet for PE, blue for PCL, and red for PEO) refer to the crystallized block at the corresponding temperature indicated on the top of the micrographs for (**a**) molten state at 118 °C, (**b**) PE at 110 °C, (**c**) PE at 50 °C, (**d**) PE and PCL at 40 °C, (**e**) PE, PCL, and PEO at 28 °C, (**f**) PE, PCL, and PEO at 24 °C, and (**g**) PE, PCL, and PEO at 0 °C.

**Figure 14:**
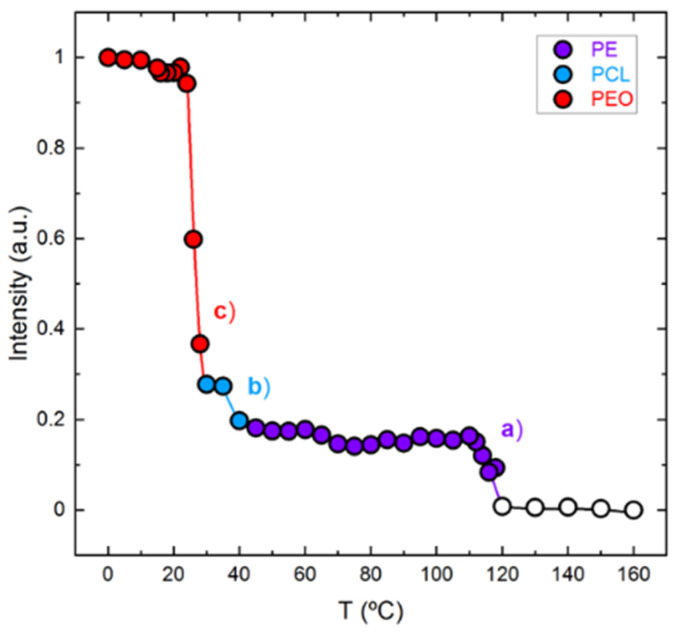
PLOM intensity measurement from data in Figure 13 as a function of temperature during cooling from the melt (20 °C/min), indicating crystallization of the following: (a) the PE block, (b) the PCL block, and (c) the PEO block for the triblock terpolymer PE_37_^9.5^-*b*-PEO_34_^8.8^-*b*-PCL_29_^7.6^ (T2). Colored data points and lines (violet for PE, blue for PCL, and red for PEO) are employed to follow the crystallization. Empty data points correspond to the molten state of the sample.

Figure 13 shows the cooling process employing as cooling rate 20 °C/min for the triblock terpolymer PE_37_^9.5^-*b*-PEO_34_^8.8^-*b*-PCL_29_^7.6^ (T2). As indicated in Figure 13a, at 118 °C, the sample is melted. Decreasing temperature to 110 °C (Figure 13b), a slight change in the micrograph indicates that the crystallization of the PE block occurred. In addition, Figure 13c shows that all PE has crystallized until saturation at 50 °C. Once again, it is challenging to notice meaningful changes in the micrographs, so the normalized intensity calculations as a function of temperature are provided in Figure 14. The first increase in intensity shows the crystallization of the PE block (curve a of Figure 14). The following slight increase in intensity corresponds to the crystallization of the PCL block (curve b of Figure 14), also shown in Figure 13d at 40 °C. Cooling down the sample, the last block to crystallize is the PEO block (Figure 13e,f), and its crystallization continues until saturation is obtained at approximately 0 °C (Figure 13g). Curve c in Figure 14 indicates that the crystallization of the PEO block starts at around 28 °C and continues with further decreases in temperature.

Figures S7 and S8 in the SI provide the subsequent heating scan and the normalized intensity measurements of the PE_37_^9.5^-*b*-PEO_34_^8.8^-*b*-PCL_29_^7.6^ (T2) triblock terpolymer, respectively. The discussed results agree well with DSC (Figure 4) and WAXS (Figure 7 and Figure 8) according to the evidences discussed above.

## 4. Conclusions

The main objective of this study is the analysis of the morphology and crystallization of triblock terpolymers with three potentially crystallizable blocks: the apolar PE and the polar PEO (biocompatible), and PCL (biodegradable) blocks, as well as their corresponding precursors. Although adding a third block to diblock copolymers makes the study more challenging, it was possible to ascertain the crystallization sequence of each of the blocks following the crystallization process by three complementary techniques: DSC, WAXS, and PLOM.

The aim of comparing two triblock terpolymers, PE_22_^7.1^-*b*-PEO_46_^15.1^-*b*-PCL_32_^10.4^ (T1) and PE_37_^9.5^-*b*-PEO_34_^8.8^-*b*-PCL_29_^7.6^ (T2), was to determine the effect of composition and molecular weight on the properties. Regarding melt miscibility, both triblock terpolymers (T1 and T2) show weak phase segregation, and the microstructure present in the melt is destroyed when crystallization of the first block starts (PE crystallization). Furthermore, the crystallization of the three blocks upon cooling from the melt employing 20 °C/min as cooling rate in both triblock terpolymers is identified. The crystallization sequence resulted as follows: the PE block crystallized first, followed by the PCL block and finally by the PEO block, as evidenced by DSC, in situ WAXS experiments, and PLOM observations with light intensity calculations.

The crystalline behavior of both triblock terpolymers (T1 and T2) is very similar regardless of the molecular weight and composition. However, for their corresponding diblock copolymer precursors, the effect of the PE block content and the molecular weight is significant. The PE_32_^7.1^-*b*-PEO_68_^15.1^ diblock copolymer is melt miscible, and the PE block crystallization is hindered due to its low content (32 wt%). Nevertheless, in the PE_52_^9.5^-*b*-PEO_48_^8.8^ diblock copolymer, the PE block crystallization is enhanced due to its higher content (52 wt%) and phase segregated nature in the melt.

The fact that three different blocks can crystallize in a triblock terpolymer forming a triple crystalline material opens a window for new applications, such as drug delivery devices. In this respect, a comprehensive understanding of these materials could be beneficial to tune their crystallizability and obtain new materials with enhanced properties.

## Figures and Tables

**Table 1:** Number-average molecular weight (*M_n_*) and polydispersity of homopolymers, diblock copolymers, and triblock terpolymers. Subscripts represent composition in wt %, and superscripts indicate *M_n_* values of each block in kg/mol.

Sample	*M_n_* PE (g/mol)	*M_n_* PEO (g/mol)	*M*_n_ PCL (g/mol)	Ɖ ^a^
PE^7.1^	7100	-	-	1.32
PE_32_^7.1^-*b*-PEO_68_^15.1^	7100	15,100	-	<1.3
PE_22_^7.1^-*b*-PEO_46_^15.1^-*b*-PCL_32_^10.4^ (**T1**)	7100	15,100	10,400	<1.3
PE^9.5^	9500	-	-	1.28
PE_52_^9.5^-*b*-PEO_48_^8.8^	9500	8800	-	<1.3
PE_37_^9.5^-*b*-PEO_34_^8.8^-*b*-PCL_29_^7.6^ (**T2**)	9500	8800	7600	<1.3

^a^ In the case of PEO-containing polymers, the polydispersity values are not correct since PEO is adsorbed by the columns used in high-temperature GPC. Using a soluble in THF terpolymer (low molecular weight PE) we were able to prove that Ɖ of the terpolymers is below 1.3 (for more details see our Ref. [103]).

## Data Availability

The present data in this research is available upon request from the corresponding author.

## Supplementary Materials

The following are available online at https://www.mdpi.com/article/10.3390/polym13183133/s1. Table S1: χ and χN values of diblock copolymers (precursors) and diblock copolymer pairs in the triblock terpolymers, calculated at 180 °C. Table S2: Thermal DSC cooling properties of the homopolymers PE, diblock copolymers PE-*b*-PEO, and triblock terpolymers PE-*b*-PEO-*b*-PCL (T1 and T2). Crystallization enthalpies are normalized according to block content. Table S3: Thermal DSC healing properties of the homopolymers PE, diblock copolymers PE-*b*-PEO, and triblock terpolymers PE-*b*-PEO-*b*-PCL (T1 and T2). Melting enthalpies are normalized according to block content in each of the samples. Table S4: Crystallinity values (%) of the samples calculated from DSC heating scans taking into account the mass fractions of each of the blocks and using X_c_ = (ΔH_m_/ΔH_m,100%_)·100 and enthalpy of fusion of 100% crystalline polymers (ΔH_m,100%_) is taken from literature: 293 J/g for PE [107], 139 J/g for PCL [108] and 214 J/g for PEO [109]. Table S5: WAXS indexation for all the samples [19,92]. Figure S1: WAXS patterns taken during subsequent heating at 20 °C/min for (a) PE^7.1^, (b) PE_32_^7.1^-*b*-PEO_68_^15.1^, and (c) PE_22_^7.1^-*b*-PEO_46_^15.1^-*b*-PCL_32_^10.4^ (T1) at different temperatures with arrows indicating transitions for each block (violet for PE, blue for PCL, and red for PEO) and the corresponding (hkl) planes of the blocks. Figure S2: Normalized WAXS intensities as a function of temperature calculated from heating WAXS data in Figure S1 with close-ups for (a) PE^7.1^, (b) PE_32_^7.1^-*b*-PEO_68_^15.1^, and (c) PE_22_^7.1^-*b*-PEO_46_^15.1^-*b*-PCL_32_^10.4^ (T1). Colored data points and lines (violet for PE, blue for PCL, and red for PEO) are employed to follow the crystallization of each block. Empty data points represent the molten state of the corresponding block in the samples. Figure S3: WAXS patterns taken during subsequent heating at 20 °C/min for (a) PE^9.5^, (b) PE_52_^9.5^-*b*-PEO_48_^8.8^, and (c) PE_37_^9.5^-*b*-PEO_34_^8.8^-*b*-PCL_29_^7.6^ (T2) at different temperatures with arrows indicating transitions for each block (violet for PE, blue for PCL and red for PEO) and the corresponding (hkl) planes of the blocks. Figure S4: Normalized WAXS intensities as a function of temperature calculated from heating WAXS data in Figure S3 for (a) PE^9.5^, (b) PE_52_^9.5^-*b*-PEO_48_^8.8^, and (c) PE_37_^9.5^-*b*-PEO_34_^8.8^-*b*-PCL_29_^7.6^ (T2). Colored data points and lines (violet for PE, blue for PCL, and red for PEO) are employed to follow the crystallization of each block. Empty data points represent the molten state of the corresponding block in the samples. Figure S5: PLOM subsequent heating micrographs from 0 °C to the melt at 20 °C/min for the triblock PE_22_^7.1^-*b*-PEO_46_^15.1^-*b*-PCL_32_^10.4^ (T1) with colored boxes indicating the crystallization of each of the blocks (violet for PE, blue for PCL and red for PEO) and the crystallized blocks in each of the micrographs for (a) PE, PCL, and PEO at 0 °C, (b) PE, PCL, and PEO at 25 °C, (c) PE, PCL, and PEO at 50 °C, (d) PE, PCL, and PEO at 70 °C, (e) PE at 72 °C, (f) PE at 125 °C, and (g) molten state at 130 °C. Figure S6: PLOM intensity measurements from micrographs of Figure S5 as a function of temperature indicating melting of the (a) PEO block, (b) PCL block, and (c) PE block for the triblock terpolymer PE_22_^7.1^-*b*-PEO_46_^15.1^-*b*-PCL_32_^10.4^ (T1) with colored data points and lines (red for PEO, blue for PCL and violet for PE) to follow the crystallization of each block. Empty data points represent the molten state of the sample. Figure S7: PLOM subsequent heating micrographs from 10 °C to the melt at 20 °C/min for the triblock PE_37_^9.5^-*b*-PEO_34_^8.8^-*b*-PCL_29_^7.6^ (T2) with colored boxes indicating the crystallization of each of the blocks (violet for PE, blue for PCL and red for PEO) and the crystallized blocks in each of the micrographs for (a) PE, PCL, and PEO at 10 °C, (b) PE, PCL, and PEO at 60 °C, (c) PE and PCL at 65 °C, (d) PE and PCL at 70 °C, (e) PE at 75 °C, (f) PE at 130 °C, (g) PE a 145 °C, and h) molten state at 150 °C. Figure S8: PLOM intensity measurements from micrographs of Figure S7 as a function of temperature indicating melting of the (a) PEO block, (b) PCL block, and (c) PE block for the triblock terpolymer PE_37_^9.5^-*b*-PEO_34_^8.8^-*b*-PCL_29_^7.6^ (T2) with colored data points and lines (red for PEO, blue for PCL and violet for PE) to follow the crystallization of each block. Empty data points represent the molten state of the sample.
