# Supplementary material for: Crystallization and Morphology of Triple Crystalline Polyethylene-b-poly(ethylene oxide)-b-poly(ε-caprolactone) PE-b-PEO-b-PCL Triblock Terpolymers
Source: Polymers (Basel). 2021 Sep 16;13(18):3133. doi: 10.3390/polym13183133 (PMC8473441; doi:10.3390/polym13183133)
Supplement: Supplementary file 1 [file polymers-13-03133-s001.zip › polymers-1386604-supplementary.pdf]

Supporting Information

Crystallization and morphology of triple crystalline PE-*b*-PEO-*b*-PCL triblock terpolymers

Eider Matxinandiarena[1], Agurtzane Múgica[1], Manoli Zubitur[2], Viko Ladelta[3], George Zapsas[3], Dario Cavallo[4], Nikos Hadjichristidis[3], Alejandro J. Müller[1,5*]*

[1]POLYMAT and Department of Polymers and Advanced Materials: Physics, Chemistry and Technology, University of the Basque Country UPV/EHU, Paseo Manuel Lardizábal 3, 20018, Donostia-San Sebastián, Spain.

[2]Department of Chemical and Environmental Engineering, University of the Basque Country, UPV/EHU, Plaza Europa 1, 20018 Donostia-San Sebastián, Spain

[3]Polymer Synthesis Laboratory, KAUST Catalysis Center, Physical Sciences and Engineering Division, King Abdullah University of Science and Technology (KAUST), Thuwal, Saudi Arabia

[4]Department of Chemistry and Industrial Chemistry, University of Genova, via Dodecaneso 31, 16146 Genova, Italy

[5]Ikerbasque, Basque Foundation for Science, Plaza Euskadi 5, 48009 Bilbao, Spain.

Corresponding Author
*email: alejandrojesus.muller@ehu.es
*email: Nikolaos.Hadjichristidis@kaust.edu.sa

Table S1. χ and χN values of diblock copolymers (precursors) and diblock copolymer pairs in the triblock terpolymers, calculated at 180 °C

Samples	Diblock pairs	χ	χN
$PE_{32}^{7.1}$-*b*-$PEO_{68}^{15.1}$	PE-*b*-PEO	0.78	136
$PE_{22}^{7.1}$-*b*-$PEO_{46}^{15.1}$-*b*-$PCL_{32}^{10.4}$	PE-*b*-PEO<br>PEO-*b*-PCL<br>PE-*b*-PCL	0.78<br>0.37<br>0.59	136<br>31<br>38
$PE_{52}^{9.5}$-*b*-$PEO_{48}^{8.8}$	PE-*b*-PEO	0.78	99
$PE_{37}^{9.5}$-*b*-$PEO_{34}^{8.8}$-*b*-$PCL_{29}^{7.6}$	PE-*b*-PEO<br>PEO-*b*-PCL<br>PE-*b*-PCL	0.78<br>0.37<br>0.59	99<br>20<br>36

Table S2. Thermal DSC cooling properties of the homopolymers PE, diblock copolymers PE-*b*-PEO, and triblock terpolymers PE-*b*-PEO-*b*-PCL (T1 and T2). Crystallization enthalpies are normalized according to block content

	$T_{c,\ PEO}$ (ºC)	$T_{c,\ PCL}$ (ºC)	$T_{c,\ PE}$ (ºC)	$\Delta H_{c,\ PEO}$ (J/g)	$\Delta H_{c,\ PCL}$ (J/g)	$\Delta H_{c,\ PE}$ (J/g)
**$PE^{7.1}$**	-	-	116.6	-	-	229.0
**$PE_{32}^{7.1}$ -*b*- $PEO_{68}^{15.1}$**	43.1	-	76.4<br>81.4<br>118.4	177.05	-	21.5
**$PE_{22}^{7.1}$ -*b*- $PEO_{46}^{15.1}$ -*b*- $PCL_{32}^{10.4}$ (T1)**	39.1*		76.5	35.4	24.6	13.5
**$PE^{9.5}$**	-	-	107.2	-	-	156.2
**$PE_{52}^{9.5}$ -*b*- $PEO_{48}^{8.8}$**	45.2	-	100.5	177.0	-	80.9
**$PE_{37}^{9.5}$ -*b*- $PEO_{34}^{8.8}$ -*b*- $PCL_{29}^{7.6}$ (T2)**	38.8*		102.5	21.0	17.9	113.4

*As crystallization temperatures of the PEO/PCL blocks are overlapped, a single T_c value is provided, and enthalpies are an estimation calculated according to block content

Table S3. Thermal DSC heating properties of the homopolymers PE, diblock copolymers PE-*b*-PEO, and triblock terpolymers PE-*b*-PEO-*b*-PCL (T1 and T2). Melting enthalpies are normalized according to block content in each of the samples

	$T_{m, PEO}$ (ºC)	$T_{m, PCL}$ (ºC)	$T_{m, PE}$ (ºC)	$\Delta H_{m, PEO}$ (J/g)	$\Delta H_{m, PCL}$ (J/g)	$\Delta H_{m, PE}$ (J/g)
**$PE^{7.1}$**	-	-	129.7	-	-	224.8
**$PE_{32}^{7.1}$ -*b*- $PEO_{68}^{15.1}$**	65.1	-	120.0<br>126.7	181.5	-	20.8
**$PE_{22}^{7.1}$ -*b*- $PEO_{46}^{15.1}$ -*b*- $PCL_{32}^{10.4}$ (T1)**	64.2*		122.0	38.7	26.9	13.1
**$PE^{9.5}$**	-	-	117.0	-	-	160.2
**$PE_{52}^{9.5}$ -*b*- $PEO_{48}^{8.8}$**	62.6	-	113.0	184.6	-	78.8
**$PE_{37}^{9.5}$ -*b*- $PEO_{34}^{8.8}$ -*b*- $PCL_{29}^{7.6}$ (T2)**	51.4	56.3	113.7	22.8	19.4	127.6

*As crystallization temperatures of the PEO and PCL blocks are overlapped, a single value is provided, and the corresponding enthalpies are an estimation calculated according to block content

Table S4. Crystallinity values (%) of the samples calculated from DSC heating scans taking into account the mass fractions of each of the blocks and using $X_c=(\Delta H_m/\ \Delta H_{m,100\%})\cdot 100$ and enthalpy of fusion of 100% crystalline polymers ($\Delta H_{m,100\%}$) is taken from literature: 293 J/g for PE[1], 139 J/g for PCL[2] and 214 J/g for PEO[3]

	$X_{c, PEO}$ (%)	$X_{c, PCL}$ (%)	$X_{c, PE}$ (%)
**$PE^{7.1}$**	-	-	76.7
**$PE_{32}^{7.1}$ -*b*- $PEO_{68}^{15.1}$**	84.8	-	7.1
**$PE_{22}^{7.1}$ -*b*- $PEO_{46}^{15.1}$ -*b*- $PCL_{32}^{10.4}$ (T1)**	39.3*	60.4*	4.5
**$PE^{9.5}$**	-	-	54.7
**$PE_{52}^{9.5}$ -*b*- $PEO_{48}^{8.8}$**	86.2	-	26.9
**$PE_{37}^{9.5}$ -*b*- $PEO_{34}^{8.8}$ -*b*- $PCL_{29}^{7.6}$ (T2)**	31.3*	48.1*	43.5

*PEO and PCL crystallinity values in the triblock terpolymers are an estimation according to block content since the melting peaks of the blocks are overlapped, and melting enthalpies cannot be distinguished properly

Table S5. WAXS indexation for all the samples[4, 5]

Blocks	(hkl) planes	q values (nm^{-1})
**PLLA**	(010)	10.3
**PLLA**	(110)/(200)	12.0
**PLLA**	(113)/(203)	13.5
**PEO**	(120)	13.8
**PE**	(110)	15.4
**PCL**	(110)	15.5
**PLLA**	(210)	15.7
**PEO**	(032)/(112)/(132)/(212)	16.4
**PCL**	(200)	16.7
**PE**	(200)	16.9

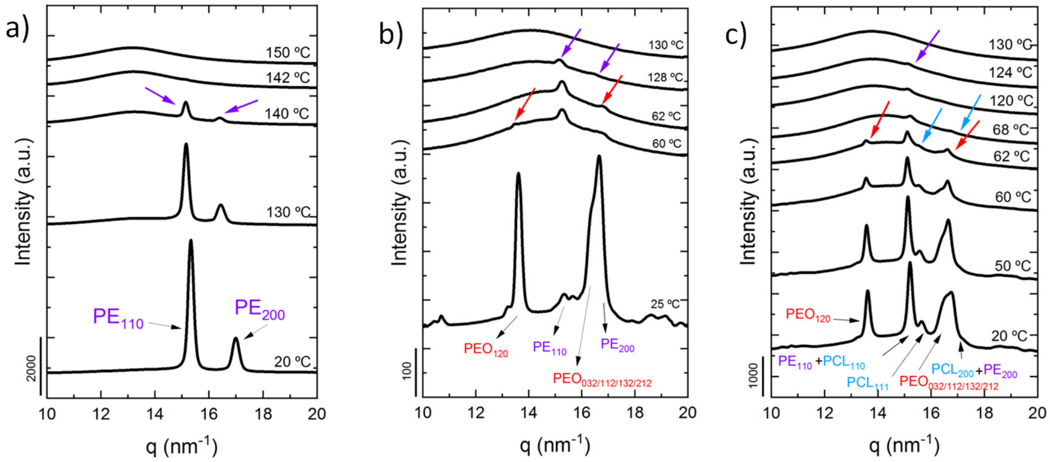

Figure S1. WAXS patterns taken during subsequent heating at 20 °C/min for a) PE$^{7.1}$, b) PE$_{32}^{7.1}$ -*b*- PEO$_{68}^{15.1}$ and c) PE$_{22}^{7.1}$ -*b*- PEO$_{46}^{15.1}$ -*b*- PCL$_{32}^{10.4}$ (T1) at different temperatures with arrows indicating transitions for each block (violet for PE, blue for PCL and red for PEO) and the corresponding (hkl) planes of the blocks

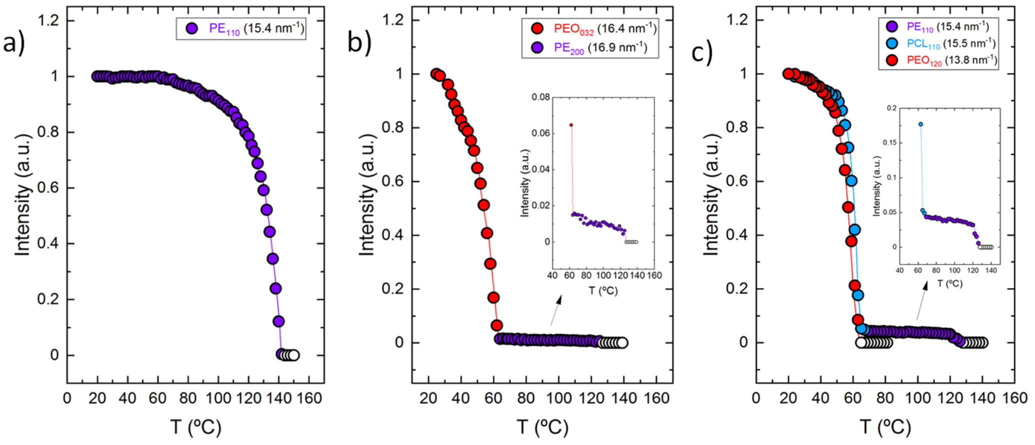

Figure S2. Normalized WAXS intensities as a function of temperature calculated from heating WAXS data in Figure S1 with close-ups for a) $PE^{7.1}$, b) $PE_{32}^{7.1}$ -*b*- $PEO_{68}^{15.1}$ and c) $PE_{22}{}^{7.1}$ -*b*- $PEO_{46}^{15.1}$ -*b*- $PCL_{32}^{10.4}$ (T1). Colored data points and lines (violet for PE, blue for PCL, and red for PEO) are employed to follow the crystallization of each block. Empty data points represent the molten state of the corresponding block in the samples

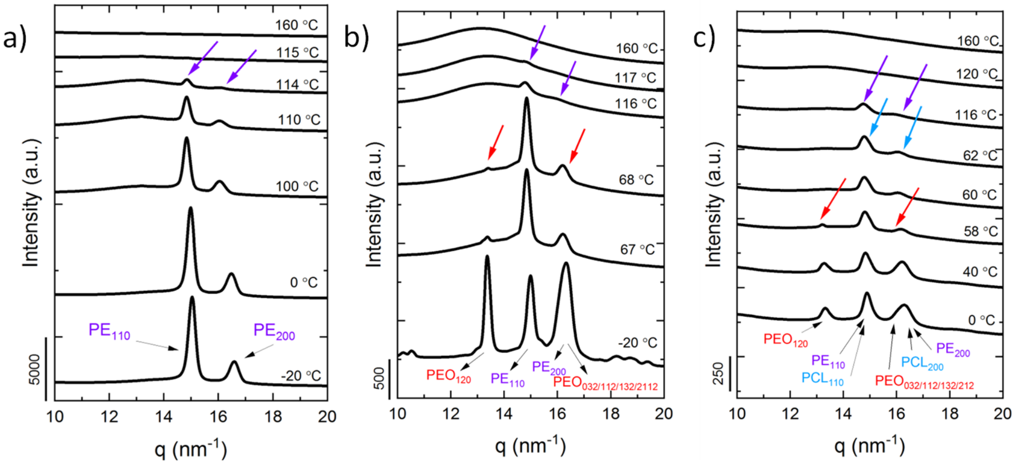

Figure S3. WAXS patterns taken during subsequent heating at 20 °C/min for a) $PE^{9.5}$, b) $PE_{52}^{9.5}$ -*b*- $PEO_{48}^{8.8}$ and c) $PE_{37}{}^{9.5}$ -*b*- $PEO_{34}^{8.8}$ -*b*- $PCL_{29}^{7.6}$ (T2) at different temperatures with arrows indicating transitions for each block (violet for PE, blue for PCL and red for PEO) and the corresponding (hkl) planes of the blocks

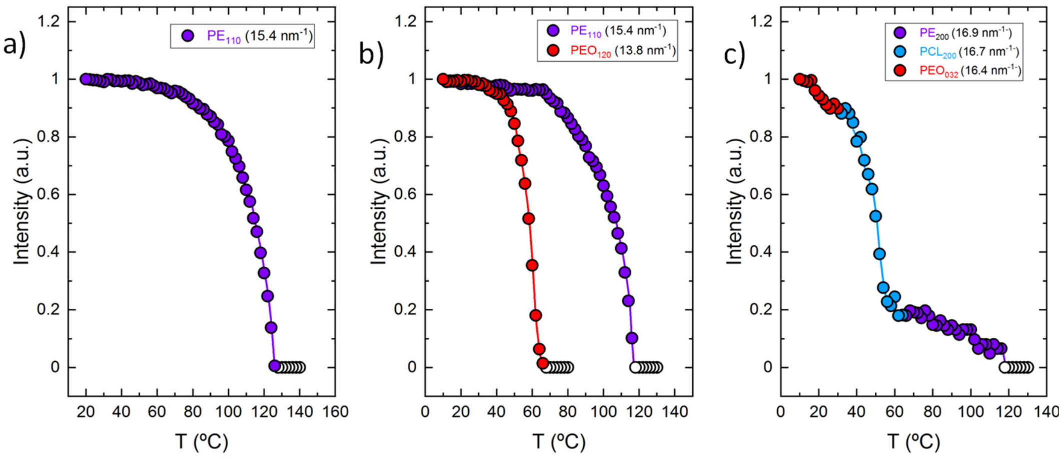

Figure S4. Normalized WAXS intensities as a function of temperature calculated from heating WAXS data in Figure S3 for a) $PE^{9.5}$, b) $PE_{52}^{9.5}$ -*b*- $PEO_{48}^{8.8}$ and c) $PE_{37}^{9.5}$ -*b*- $PEO_{34}^{8.8}$ -*b*- $PCL_{29}^{7.6}$ (T2). Colored data points and lines (violet for PE, blue for PCL, and red for PEO) are employed to follow the crystallization of each block. Empty data points represent the molten state of the corresponding block in the samples

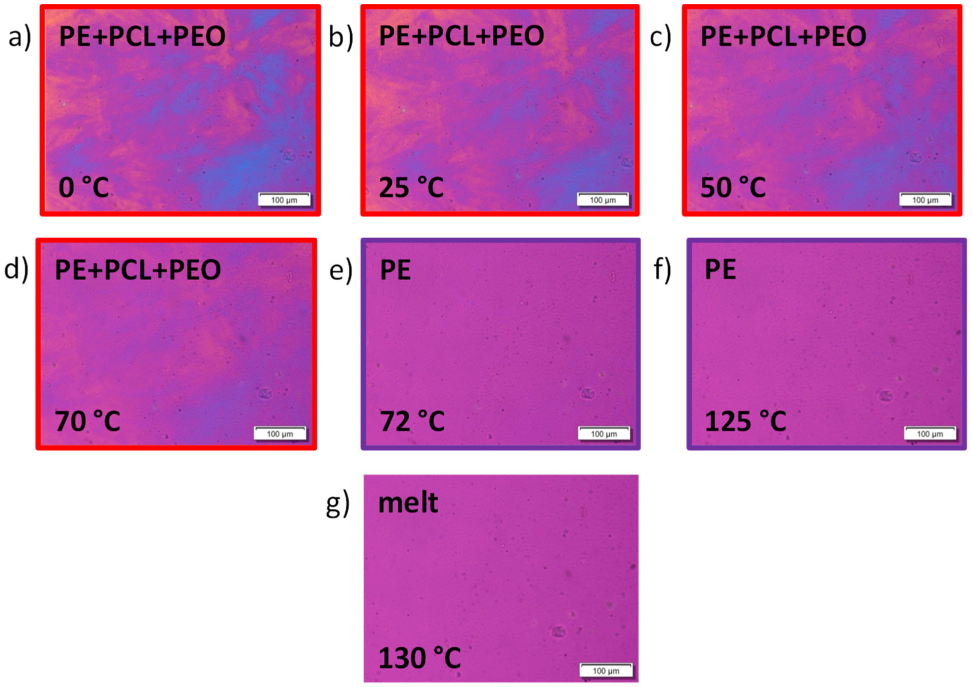

Figure S5. PLOM subsequent heating micrographs from 0 °C to the melt at 20 °C/min for the triblock $PE_{22}^{7.1}$ -*b*- $PEO_{46}^{15.1}$ -*b*- $PCL_{32}^{10.4}$ (T1) with colored boxes indicating the crystallization of each of the blocks (violet for PE, blue for PCL and red for PEO) and the crystallized blocks in each of the micrographs for a) PE, PCL and PEO at 0 °C, b) PE, PCL and PEO at 25 °C, c) PE, PCL and PEO at 50 °C, d) PE, PCL and PEO at 70 °C, e) PE at 72 °C, f) PE at 125 °C, and g) molten state at 130 °C

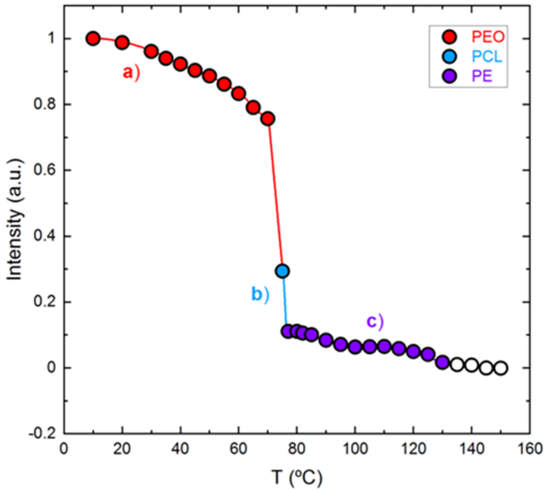

Figure S6. PLOM intensity measurements from micrographs of Figure S5 as a function of temperature indicating melting of the a) PEO block, b) PCL block, and c) PE block for the triblock terpolymer $PE_{22}^{7.1}$ -*b*- $PEO_{46}^{15.1}$ -*b*- $PCL_{32}^{10.4}$ (T1) with colored data points and lines (red for PEO, blue for PCL and violet for PE) to follow the crystallization of each block. Empty data points represent the molten state of the sample.

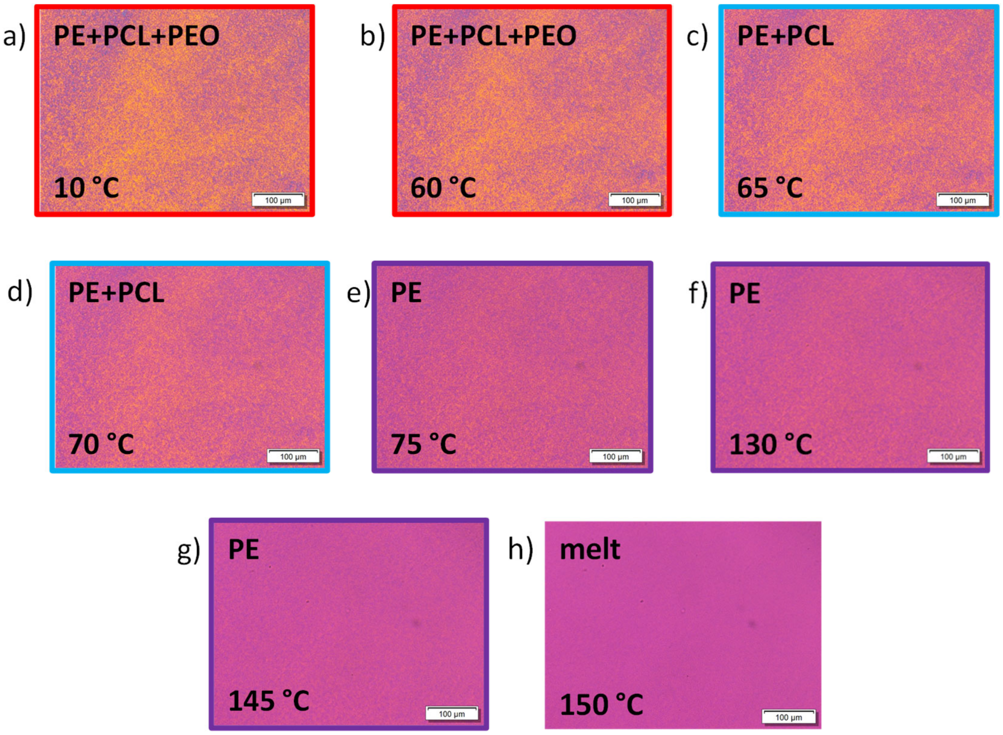

Figure S7. PLOM subsequent heating micrographs from 10 °C to the melt at 20 °C/min for the triblock $PE_{37}{}^{9.5}$ -*b*- $PEO_{34}{}^{8.8}$ -*b*- $PCL_{29}{}^{7.6}$ (T2) with colored boxes indicating the crystallization of each of the blocks (violet for PE, blue for PCL and red for PEO) and the crystallized blocks in each of the micrographs for a) PE, PCL, and PEO at 10 °C, b) PE, PCL, and PEO at 60 °C, c) PE and PCL at 65 °C, d) PE and PCL at 70 °C, e) PE at 75 °C, f) PE at 130 °C, g) PE a 145 °C, and h) molten state at 150 °C

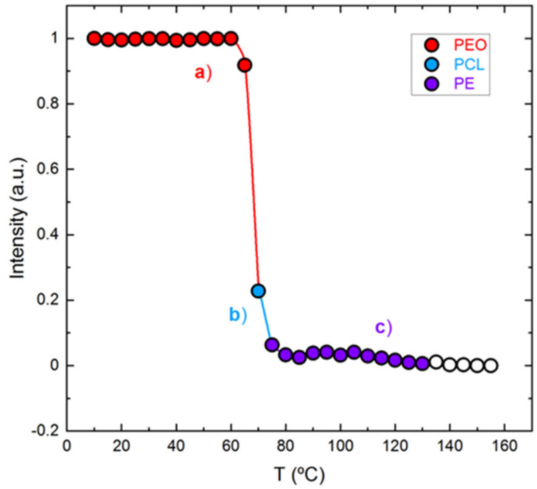

Figure S8. PLOM intensity measurements from micrographs of Figure S7 as a function of temperature indicating melting of the a) PEO block, b) PCL block, and c) PE block for the triblock terpolymer $PE_{37}{}^{9.5}$ -*b*- $PEO_{34}{}^{8.8}$ -*b*- $PCL_{29}{}^{7.6}$ (T2) with colored data points and lines (red for PEO, blue for PCL and violet for PE) to follow the crystallization of each block. Empty data points represent the molten state of the sample.

REFERENCES

1. Ren, M.; Tang, Y.; Gao, D.; Ren, Y.; Yao, X.; Shi, H.; Zhang, T.; Wu, C., Recrystallization of biaxially oriented polyethylene film from partially melted state within crystallite networks. *Polymer* **2020,** *191*, 122291.

2. Izquierdo, R. G.-G., N.; Rodríguez, M. T.; Cáceres, E.; García, S. J.; Gómez Ribelles, J. M.; Monleón, M.; Monllau, J. C.; Suay, J., Biodegradable PCL scaffolds with an interconnected spherical pore network for tissue engineering. *Journal of Biomedical Materials Research* **2007**, 25-35.

3. Ebers, L. S.; Auvergne, R.; Boutevin, B.; Laborie, M. P., Impact of PEO structure and formulation on the properties of a Lignin/PEO blend. *Industrial Crops and Products* **2020,** *143*, 111883.

4. Palacios, J. K.; Mugica, A.; Zubitur, M.; Iturrospe, A.; Arbe, A.; Liu, G.; Wang, D.; Zhao, J.; Hadjichristidis, N.; Müller, A. J., Sequential crystallization and morphology of triple

crystalline biodegradable PEO-*b*-PCL-*b*-PLLA triblock terpolymers. *Royal Society of Chemistry Adv.* **2016,** *6*, 4739.

5. Castillo, R. V.; Müller, A. J.; Lin, M. C.; Chen, H. L.; Jeng, U. S.; Hillmyer, M. A., Confined crystallization and morphology of melt segregated PLLA-*b*-PE and PLDA-*b*-PE diblock copolymers. *Macromolecules* **2012,** *45*, 4254-4261.
